# Supportive interventions to improve retention on ART in people with HIV in low- and middle-income countries: A systematic review

**DOI:** 10.1371/journal.pone.0208814

**Published:** 2018-12-14

**Authors:** Amy W. Penn, Hana Azman, Hacsi Horvath, Kelly D. Taylor, Matthew D. Hickey, Jay Rajan, Eyerusalem K. Negussie, Margaret Doherty, George W. Rutherford

**Affiliations:** 1 School of Medicine, University of California, San Francisco, San Francisco, California, United States of America; 2 Institute for Global Health Sciences, University of California, San Francisco, San Francisco, California, United States of America; 3 Department of HIV/AIDS, World Health Organization, Geneva, Switzerland; Azienda Ospedaliera Universitaria di Perugia, ITALY

## Abstract

**Objectives:**

To determine whether supportive interventions can increase retention in care for patients on antiretroviral therapy (ART) in low- and middle-income countries (LMIC).

**Design:**

Systematic review and meta-analysis.

**Methods:**

We used Cochrane Collaboration methods. We included randomised controlled trials (RCT) and observational studies with comparators conducted in LMIC. Our principal outcomes were retention, mortality and the combined outcome of lost-to-follow-up (LTFU) or death.

**Results:**

We identified seven studies (published in nine articles); six of the studies were from Sub-Saharan Africa. We found four types of interventions: 1) directly observed therapy plus extra support (“DOT-plus”), 2) community-based adherence support, 3) adherence clubs and 4) extra care for patients with low CD4 count. One RCT of a community-based intervention showed significantly improved retention at 12 months (RR 1.14, 95% CI 1.02 to 1.27), and three observational studies found significantly improved retention for paediatric patients followed for 12 to 36 months (RR 1.07, 95 CI 1.03 to 1.11), and for adult patients at 12 (RR 1.38, 95% CI 1.13 to 1.70) and 60 months (RR 1.07, 95% CI 1.07 to 1.08). One observational study of adherence clubs showed significantly reduced LTFU or mortality (RR 0.20, 95% CI 0.12 to 0.33). A cluster RCT of an extra-care intervention for high-risk patients also showed a significant increase in retention (RR 1.06, 95% CI 1.01 to 1.10), and an observational study of extra nursing care found a significant decrease in LTFU or mortality (RR 0.76, 95% CI 0.66 to 0.87).

**Conclusions:**

Supportive interventions are associated with increased ART programme retention, but evidence quality is generally low to moderate. The data from this review suggest that programmes addressing psychosocial needs can significantly help retain patients in care.

## Introduction

Globally, approximately 36.7 million people were living with human immunodeficiency virus (HIV) infection at the end of 2016, and, as of June 2017, nearly 20.9 million people were estimated to be on antiretroviral therapy (ART) [1]. ART has been associated with marked reductions in mortality at the population level regarding AIDS-associated opportunistic infections and malignancies, [2] and a high prevalence of viral suppression to reduce HIV transmission [3]. In order for ART to be effective, it is essential that patients maintain very high levels of adherence. Adherence to ART is important not only for the patient's survival but also to prevent drug resistance with resultant treatment failure and the need to switch to second-line ART regimens, which are often more expensive and complex than first-line regimens [4, 5]. Another benefit of adherence is that patients with a suppressed viral load are less likely to transmit HIV to sexual partners and perinatally [6]. It is thus crucial that patients diagnosed with HIV should enter medical care as soon as possible, be retained on lifelong ART and adhere to treatment [7].

A systematic review (and a follow-up review) of studies published or presented between 2002 and 2009 found that ART programmes in sub-Saharan Africa retained an average of about 80% of patients after six months, about 70% after two years and about 65% after three years [8, 9]. Among adults, reported retention rates were about 78%, 71%, and 69% after one, two, and three years on ART, respectively, with slightly better retention among adolescents at about 85%, 81%, and 81% [10, 11]. Two Option B+ programme studies in sub-Saharan Africa for the prevention of mother to child transmission (PMTCT) found 12% to 17% of mothers were lost to follow up (LTFU) within six months of ART initiation [12, 13] while an observational cohort study of PMTCT in Malawi found that, retention was 77%, 71%, and 69% one, two, and three years after ART initiation [14].

### Objective

Our objective was to review the scientific literature systematically and assess the efficacy and effectiveness of supportive interventions, both community-based and clinic-based, for promoting adherence or retention in care for people with HIV infection in low- and middle-income countries (LMIC) who have initiated ART.

## Materials and methods

We included randomised controlled trials (RCT), non-randomised studies and observational studies conducted in LMIC that used standard of care as the comparator. We used the World Bank definition of LMIC for study searching [15]. Participants include HIV-infected persons of all ages residing in LMIC. We included any intervention specifically designed to improve adherence or retention in care for people with HIV infection who had initiated ART and that had an outcome of retention in care. Because these interventions featured peer-, community-, and clinic-based support beyond standard care, we refer to them as “supportive interventions”. The unit of analysis was the individual patient.

We excluded: 1) studies that evaluated interventions concerned with linkage to and retention in care in the period of time between HIV diagnosis and ART initiation, 2) decentralisation of care interventions (covered in a Cochrane review [16]), and 3) task-shifting interventions from physician to non-physician (also covered in a Cochrane review [17]) and from pharmacist to non-pharmacist [18] The rationale for excluding these types of studies was to focus on interventions that aim to address life-long retention and that aim to give more interpersonal support or attention than the standard care that decentralisation or task-shifting alone would provide.

### Variables

#### Primary outcomes

Our primary outcome was retention in care following ART initiation. We defined retention in care as a patient who is still on ART (assessed at intervals longer than six months post-initiation) and has not died, transferred out, stopped treatment or been lost-to follow-up (LTFU). We also used the complement of attrition (1 minus attrition) as in indicator of retention if attrition was mentioned but did not specify the inclusion of transfer outs.

#### Secondary outcomes

Our secondary outcomes were mortality, LTFU alone, and the combined outcome of LTFU or death.

#### Standardization of outcomes

We standardised reported outcomes. Where papers reported data for a composite outcome of "loss to follow-up or death" (LTFU/death), we calculated the mathematical complement of that outcome data to estimate retention in ART programs. To illustrate, had a paper reported LTFU/death in 100 (10%) of 1000 participants in an ART program arm, we would have calculated that 900 (90%) of 1000 participants were retained in care in that arm.

### Review protocol

The original review protocol was registered in PROSPERO, the International Prospective Register of Systematic Reviews on 22 February 2015. The registration number is 17017 and can be found online: http://www.crd.york.ac.uk/PROSPERO/display_record.php?ID=CRD42015017017.

### Search methods for identification of studies

We formulated a comprehensive and exhaustive search strategy in an attempt to identify all relevant studies regardless of language or publication status (published, unpublished, in press and in progress).

We searched the following electronic databases, in the period from 1 January 1996 to the final search date (4 April 2017):

- CENTRAL (Cochrane Central Register of Controlled Trials)
- PsycINFO
- PubMed
- Web of Science
- World Health Organization (WHO) Global Health Library, which includes references from AIM (African Regional Office), LILACS (Pan American Health Organization), IMEMR (Eastern Mediterranean Regional Office), IMSEAR (South and Southeast Asia Regional Office), and WPRIM (Western Pacific Regional office).

Along with appropriate medical subject heading (MeSH) terms and relevant keywords, we used the Cochrane Highly Sensitive Search Strategy for identifying reports of RCTs in MEDLINE (Higgins 2008). The search strategy was iterative, in that references of included studies were searched for additional references. Studies published in any language were eligible for inclusion. We also contacted experts in the field to learn of any studies we may have missed.

See S1 Table for our core PubMed search strategy, which was modified and adapted as needed for use in the other databases.

#### Conference databases and ongoing trials

We searched conference abstract archives for the International AIDS Conference (IAC), and the International AIDS Society (IAS) Conference on HIV Pathogenesis, Treatment and Prevention, for abstracts presented at all conferences from 1996 through 2014. Because of internal problems with the abstract archive for the Conference on Retroviruses and Opportunity Infections (CROI), we were only able to search CROI’s abstracts for the years 2014 and 2015. We searched clinicaltrials.gov and WHO’s International Clinical Trials Registry Platform to identify ongoing trials.

### Data collection and analysis

Our methodology for data collection and analysis was based on guidance in the *Cochrane Handbook of Systematic Reviews of Interventions* [19].

#### Selection of studies

We imported search results into bibliographic citation management software (EndNote X6, Thomas Reuters, Philadelphia, USA) and excluded duplicate references that were clearly irrelevant. Three authors working independently then reviewed the titles, abstracts and descriptor terms of the remaining citations to identify potentially eligible reports (AWP, HA, JR). We obtained full text articles for all references identified as potentially meeting our inclusion criteria. Two authors working independently reviewed these full text articles and applied the inclusion criteria to establish each study's final eligibility or ineligibility (AWP with either HA or JR). Studies were reviewed for relevance based on study design, type of intervention, participants and outcome measures. Disagreements were resolved by discussion and, if necessary, a neutral third party arbiter.

#### Data extraction and management

After identifying trials for inclusion, three authors working independently examined and extracted data from each study (AWP, HA, JR). Three authors separately entered these data into standardised data extraction forms. Extracted information included study design characteristics, participant details, intervention details, outcome details and details necessary for bias risk assessment. We compared extracted data in the forms and resolved any differences by discussion.

#### Assessment of risk of bias in included studies

We used the Cochrane Collaboration tool [19] for assessing the risk of bias for each individual study and present results in summary tables. For RCTs, the Cochrane tool assesses risk of bias in individual studies across seven domains: sequence generation, allocation concealment, blinding of participants and personnel, blinding of outcome assessment, incomplete outcome data, selective outcome reporting and other potential biases. For observational studies, we assessed the risk of bias by evaluating study rigor on a 9-point scale based on systematic reviews of HIV behavioral interventions by the Johns Hopkins WHO Synthesizing Intervention Effectiveness project [20, 21] and a systematic review on HIV interventions linked with sexual and reproductive health [22]. Our assessment of bias is provided in S1 File.

#### Quality of evidence

In addition to assessing risk of bias with the Cochrane tool, we graded the quality of evidence by outcome using the Grades of Recommendation Assessment, Development and Evaluation (GRADE) approach [23]. We used GRADEpro software version 3.2 to perform our analyses (GRADEpro, GRADE Working Group, Hamilton, Canada, 2008). GRADE ranks the quality of evidence on four levels: "high," "moderate," "low" and "very low." Evidence from RCTs starts at "high," but can be downgraded based on study limitations, inconsistency of results, indirectness of evidence, imprecision or for reporting bias. Evidence from observational studies starts at "low" but can be upgraded if the magnitude of treatment effect is very large, if there is a significant dose-response relation or if all possible confounders would decrease the magnitude of an apparent treatment effect [23]. Evidence from observational studies can also be downgraded. Brief GRADE summaries are provided in S2 File.

#### Measures of treatment effect

We used Review Manager 5.2 (RevMan 2013, Cochrane Collaboration, London, UK) for preparing the review and for statistical analysis. We summarised dichotomous outcomes for effect using risk ratios (RR) with 95% confidence intervals (CI). We calculated summary statistics (using meta-analytic methods when appropriate) and present findings with regard to evidence quality in GRADE evidence profiles, for all outcomes of interest. Where populations, interventions, outcomes and outcome assessment time points were sufficiently similar, we performed meta-analyses by pooling outcome data using random effects models to calculate Mantel-Haenszel risk ratios.

#### Dealing with missing data

When outcome data were only presented in terms of proportions or percentages, we back-calculated to determine actual numerators. When studies did not report retention directly, we inferred patient retention by taking the complement of attrition (one minus attrition). We defined attrition as the sum of patients who died (of any cause) or were lost-to-follow-up.

#### Assessment of heterogeneity

We assessed statistical heterogeneity using the I^2^ statistic. Although we identified substantial heterogeneity in pooled data, we did not conduct subgroup or sensitivity analysis because we did not combine more than two studies in any given analysis.

#### Assessment of reporting biases

Where we suspected reporting bias we contacted study authors and asked them to provide missing outcome data. We attempted to minimise the potential for publication bias through our comprehensive search strategy that included evaluating published and unpublished literature, with all languages eligible.

## Result

### Results of the search

**Fig 1:**
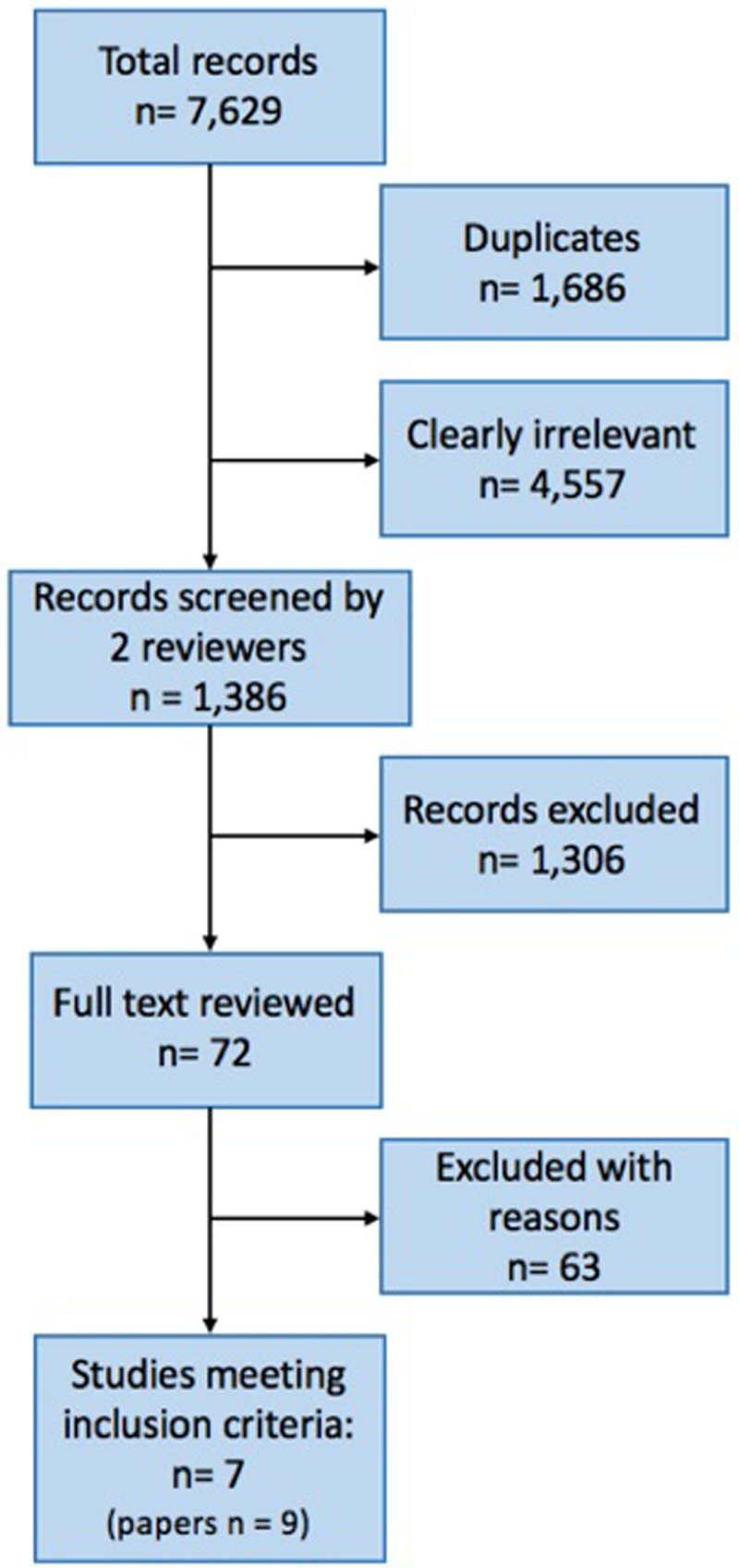
Flowchart depicting screening process.

We conducted the searches on 22 April 2014, 27 April 2015 and 4 April 2017. The searches yielded 7,629 records. After removing 1,686 duplicate records, one author (HH) screened titles and abstracts and excluded 4,557 clearly irrelevant records. We obtained the full texts of 72 references to make final determinations of whether these studies met inclusion criteria. Seven studies (nine papers) met our inclusion criteria (Fig 1). The included studies that were conducted in sub-Saharan Africa (Kenya, Mozambique, Rwanda, and South Africa) and Latin America (Peru). Only one of the six studies was an RCT [24]. Study characteristics are summarised in Table 1.

**Table 1:** Characteristics of included studies.

Study	Design	Setting & years	Participants	Number of participants	Intervention & comparator	Outcomes assessed
Braitstein 2012	Cohort (retrospective)	Kenya, 2007–2009	Adults with CD4 <100 cells/μL	4,958	Extra care for high risk patients vs. usual care	Mortality, LTFU, LTFU or died^a^
Muñoz 2010 Muñoz 2011 (CASA)	Cohort (prospective)	Peru, 2005–2007	Adults initiating ART	120	DOT-plus vs. usual care	Retention, mortality
Franke 2013	Cohort (prospective)	Rwanda, 2007–2008	Adults initiating ART	610	DOT-plus vs. usual care	Retention, mortality, LTFU
Fatti 2012 Grimwood 2012 (Kheth’Impilo)	Cohort (prospective)	South Africa, 2004–2010	Adults and children initiating ART	70,516 (66,953 adults, 3,563 children)	Community-based adherence support vs. usual care	Retention, mortality, LTFU
Luque-Fernandez 2013	Cohort (retrospective)	South Africa, 2007–2011	Adults, stable on ART	2,829	Adherence clubs: Monthly clinic-based patient support meetings vs. usual care	LTFU or died^a^
Mfinanga 2015	Cluster RCT	Tanzania, Zambia 2012–2014	Adults initiating ART with CD <200 cells/μL	1,999	Extra care for high risk patients vs. usual care	Mortality, LTFU
Pearson 2007	RCT	Mozambique 2004–2006	Adults initiating ART	350	DOT-plus vs. usual care	Retention

^a^We assessed the complement of “LTFU or died” outcomes as retention

### Effects of interventions

There were four types of supportive interventions in the included studies: directly observed therapy plus extra support (“DOT-plus”), community-based adherence support, adherence clubs and extra clinic-based supportive care for patients at high risk of non-adherence. Table 2 shows the effect estimate for all indicators and their follow-up in all included studies, which uses the standardization of the retention outcome method mentioned in the Methods section.

**Table 2:** Effect estimates per study for all measured outcomes in all Included studies with follow-up time.

Study	Assessed (months)	Risk Ratio
**RETENTION OUTCOME**		
Braitstein 2012	10	1.14 (95% CI 1.08 to 1.20)
CASA (Muñoz 2010)	12	1.38 (95% CI 1.13 to 1.70)
Franke 2013	12	1.06 (95% CI 1.00 to 1.11)
Mfinanga 2015	12	1.06 (95% CI 1.01 to 1.10)
Pearson 2007	12	1.14 (95% CI 1.02 to 1.17)
CASA (Muñoz 2011)	24	1.68 (95% CI 1.29 to 2.18)
Kheth'Impilo (Grimwood 2012)	36	1.07 (95% CI 1.03 to 1.11)
Luque-Fernandez 2013	40	1.14 (95% CI 1.11 to 1.17)
Kheth'Impilo (Fatti 2012)	60	1.07 (95% CI 1.07 to 1.08)
**MORTALITY OUTCOME**		
Braitstein 2012	10	0.69 (95% CI 0.50 to 0.94)
Franke 2013	12	0.59 (95% CI 0.31 to 1.16)
Mfinanga 2015	12	0.74 (95% CI 0.60 to 0.91)
CASA (Muñoz 2011)	24	0.35 (95% CI 0.15 to 0.83)
Kheth'Impilo (Grimwood 2012)	36	0.46 (95% CI 0.26 to 0.82)
Kheth'Impilo (Fatti 2012)	60	0.85 (95% CI 0.81 to 0.89)
**LTFU or DEATH OUTCOME**		
Braitstein 2012	10	0.76 (95% CI 0.66 to 0.87)
CASA (Muñoz 2010)	12	0.29 (95% CI 0.12 to 0.66)
Franke 2013	12	0.50 (95% CI 0.28 to 0.90)
Mfinanga 2015	12	0.78 (95% CI 0.64 to 0.94)
Pearson 2007	12	0.60 (95% CI 0.39 to 0.92)
CASA (Muñoz 2011)	24	0.28 (95% CI 0.14 to 0.55)
Luque-Fernandez 2013	40	0.20 (95% CI 0.12 to 0.33)
Kheth'Impilo (combined Fatti 2012, Grimwood 2012)	36, 60	0.81 (95% CI 0.78 to 0.83)
**LTFU OUTCOME**		
Braitstein 2012	10	0.78 (95% CI 0.67 to 0.92)
Franke 2013	12	0.30 (95% CI 0.08 to 1.09)
Mfinanga 2015	12	1.04 (95% CI 0.60 to 1.81)
Kheth'Impilo (Grimwood 2012)	36	0.82 (95% CI 0.50 to 1.35)
Kheth'Impilo (Fatti 2012)	60	0.75 (95% CI 0.72 to 0.78)

#### 1) “DOT-plus”

**Fig 2:**
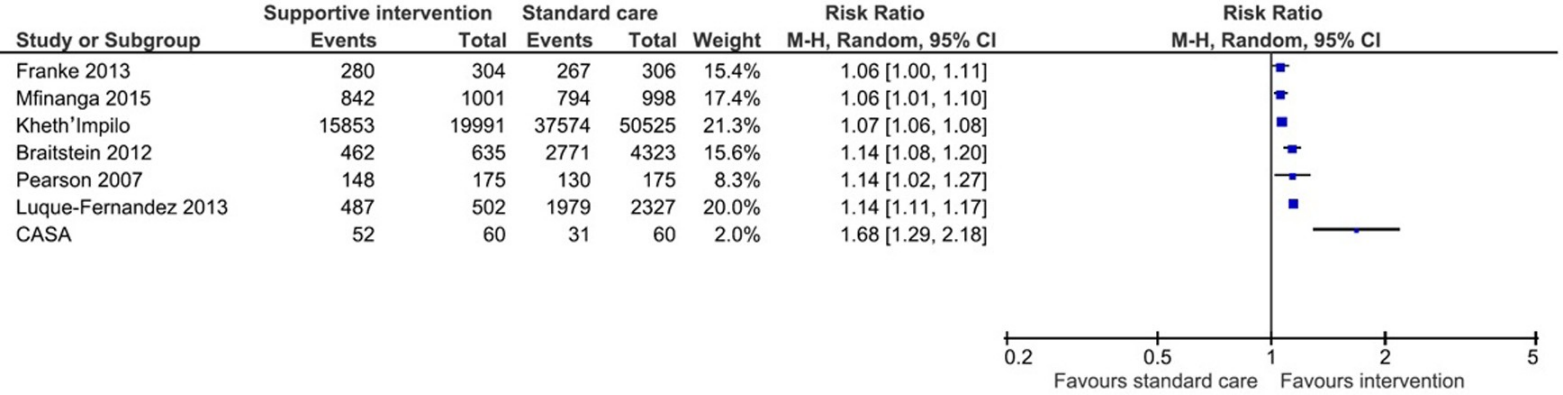
Forest plot of retention outcome (10–60 months), data unpooled. Events represent number of patients retained in care at the end of the study period.

These packages of care include peer patient advocate or treatment-supporter interventions, providing adherence and psychosocial support in addition to DOT. In the one included RCT, Pearson and colleagues in Mozambique randomised 350 ART-naïve patients to peer-delivered modified directly observed therapy (mDOT) or standard care [24]. In the Pearson study, there was a significant difference in retention at 12 months favouring the clinic-based peer mDOT intervention group (RR 1.14, 95% CI 1.02 to 1.27, Fig 2.).

**Fig 3:**
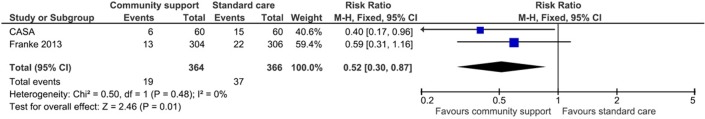
Forest plot of mortality (12 months), data pooled. Events represent number of patients who died at the end of 12 months.

**Fig 4:**
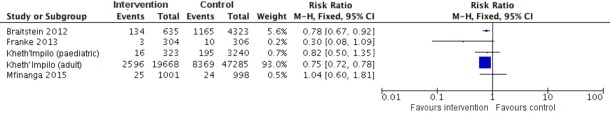
Forest plot of lost to follow-up outcome (10–60 months), data unpooled. Events represent number of patients who were were lost-to-follow-up (neither retained nor died) at the end of the study period.

**Fig 5:**
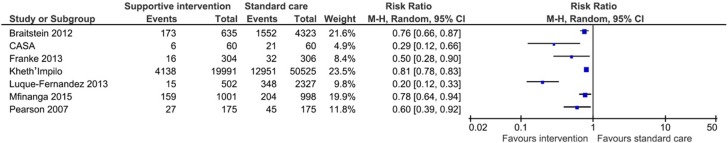
Forest plot of lost to follow-up outcome or death (12 months), data unpooled. Events represent number of patients who were were lost-to-follow-up and died at the end of the study period.

**Fig 6:**
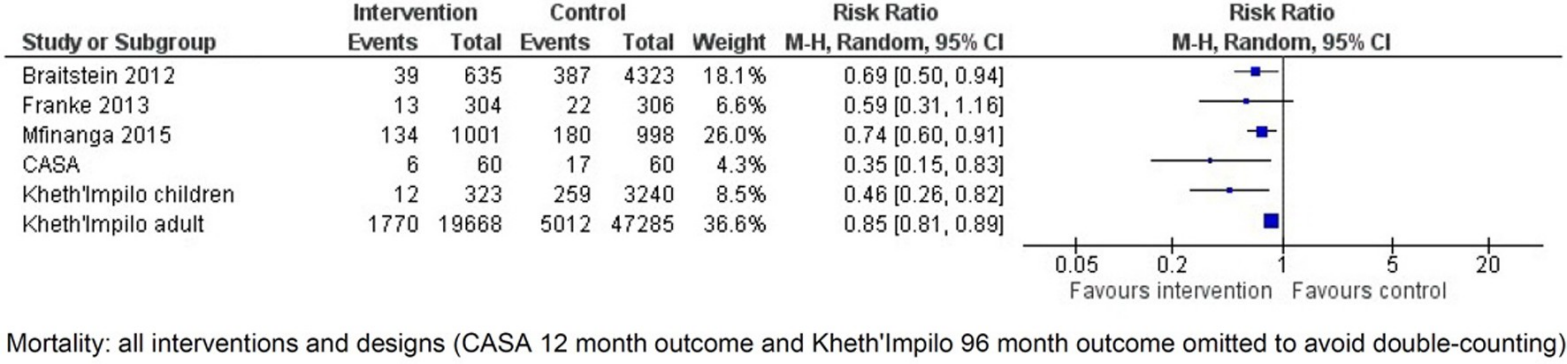
Forest plot of mortality (10–60 months), data unpooled. Events represent number of patients who died at the end of the study period.

**Fig 7:**
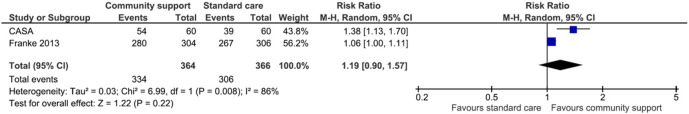
Forest plot of retention outcome (12 months), data pooled. Events represent number of patients retained in care at the end of 12 months.

Franke and colleagues conducted a prospective cohort study in Rwanda investigating the effects of having a community-based peer treatment supporter perform daily DOT at home [25]. At 12 months, there were no significant differences in retention outcome (RR 1.06, 95% CI 1.00 to 1.11, Fig 2) or 12-month mortality (RR 0.59, 95% CI 0.31 to 1.16, Fig 3) [25]. Similarly, there was no difference in LTFU alone (RR 0.30, 95% CI 0.08 to 1.09, Fig 4) [25] but there was a significantly reduced rate of LTFU or death (RR 0.50, 95% CI 0.28 to 0.90, Fig 5). Muñoz and colleagues conducted a prospective cohort study, called the Community-based Accompaniment with Supervised Antiretrovirals (CASA) study, in Peru, which employed a multi-component community-based care intervention with DOT [26, 27]. This intervention was significantly associated with increased retention at both 12 months (RR 1.38, 95% CI 1.13 to 1.70, Fig 2) and 24 months (RR 1.68, 95% CI 1.29 to 2.18, Table 2) [26, 27] and a significant reduction in 12-month (RR 0.40, 95% CI 0.17 to 0.96, Fig 3) and 24-month mortality (RR 0.35, 95% CI 0.15 to 0.83, Fig 6) [26, 27]. When the data were pooled for Franke and CASA, there was a significant increase in retention at 12 months (RR 1.19, 95% CI 0.90 to 1.57, Fig 7) and a significant reduction in 12-month (RR 0.52, 95% CI 0.30 to 0.87, Fig 3) [25, 26]. The quality of evidence for this body of literature is moderate for retention at 12 months among adults using clinic-based peer mDOT [24], while the quality of evidence for increased retention and decreased mortality and LTFU in the observational cohort studies is low [25–27].

#### 2). Community-based adherence support

We identified two cohort studies that evaluated a community-based adherence support intervention. Fatti and Grimwood separately published results from the Kheth’Impilo study, a large prospective cohort study of a community-based peer patient advocate intervention in paediatric (median age of 6.3 years, all children were under 16 years of age) [28] and adult (median age of 35.1 years, adults were defined as 16 years of age or older) [29] populations in South Africa. In Fatti and Grimwood, paid patient advocates (clinic-linked, lay healthcare personnel) trained in HIV and tuberculosis management and psychosocial support conducted home visits to supervise medication administration, perform adherence checks, counsel on barriers to adherence and refer patients to clinics if necessary. Home visits occurred weekly for the first month, and afterward would generally occur at least once monthly in Grimwood or every three months in Fatti.

***Adult cohort*.** Community-based adherence support was significantly associated with improved patient retention in the Kheth’Impilo adult cohort at 60 months (RR 1.07, 95% CI 1.07 to 1.08, Table 2) [29]. Mortality was significantly decreased in the Kheth’Impilo adult cohort at 60 months (RR 0.85, 95% CI 0.81 to 0.89, Fig 6) [29]. LTFU was reduced in the Kheth’Impilo cohort at 60 months (RR 0.75, 95% CI 0.72 to 0.78, Fig 4).

***Paediatric cohort*.** Mortality was significantly reduced for the Kheth’Impilo paediatric cohort at 36 months (RR 0.46 95% CI 0.26 to 0.82, Fig 6) [28]. The intervention was significantly associated with improved patient retention in the paediatric cohort followed at both 12 and 36 months (RR 1.07, 95% CI 1.03 to 1.11, Table 2). However, LTFU was not significantly different between those who received the intervention and those who did not at 36 months (RR 0.82, 95% CI 0.5 to 1.35, Fig 4).

There is consistent evidence that multifaceted community-based trials improved retention outcomes among adults as evaluated by observational studies. For community-based interventions targeting adherence in children, there was moderate quality evidence (upgraded for large effect size) for reduction in mortality and low quality evidence for improvement in retention. Brief GRADE summaries for each intervention type are shown in S2 File.

#### 3) Adherence clubs

Luque-Fernandez and colleagues conducted a retrospective cohort study in South Africa that compared outcomes of patients who joined facility-based adherence clubs with those who remained in standard care. Patients stable on ART for 18 months were invited to join these clubs [30]. Membership in adherence clubs was associated with significantly improved retention (RR 1.14, 95% CI 1.11 to 1.17, Fig 2) and significantly reduced LTFU or death at 40 months (RR 0.2, 95% CI 0.12 to 0.33, Fig 5).

The quality of evidence for this intervention was of moderate quality for the LTFU outcome, which was upgraded due to large effect.

#### 4) Extra community-based care for high-risk patients

Mfinanga and colleagues in Tanzania and Zambia randomised clusters of patients with <200 CD4 cells/μL at initiation of ART to either community support in addition to standard clinic or to standard care alone [31]. Extra community-based care included screening patients for cryptococcal meningitis and offering treatment if the results were positive and then weekly home visits by lay workers to deliver ART, provide adherence support and monitor side effects. At 12 months, the retention was significantly higher in the intervention arm (RR 1.06, 95% CI 1.01 to 1.10, Fig 2) and mortality significantly lower (RR 0.74, 95% CI 0.60 to 0.91, Fig 6). The combined outcome, LTFU or mortality (RR 0.78, 95% CI 0.64 to 0.94, Fig 5), was also both signtificantly reduced in the intervention arm.

Braitstein and colleagues retrospectively evaluated more frequent nurse monitoring, weekly or biweekly, in clinic for patients (with low CD4 count at ART initiation) in Kenya [32]. There was significantly improved retention (RR 1.14, 95% CI 1.08 to 1.20, Fig 2) and significantly decreased mortality (RR 0.69, 95% CI 0.5 to 0.94, Fig 6) and LTFU (RR 0.78, 95% CI 0.67 to 0.92, Fig 4) at a median 318 days among patients exposed to more frequent nurse monitoring. This was a significant decrease in the proportion with the combined outcome of death or LTFU (RR 0.76, 95% CI 0.66 to 0.87, Fig 5) among those exposed.

The quality of evidence for this intervention was very low for both the mortality and LTFU outcomes. The quality was downgraded because of the retrospective nature of the analysis.

It would be ideal to have longer follow-up in studies, such as three years or greater, to more accurately reflect retention in care over time. Only three of nine articles studied outcomes at three years or more, including Grimwood 2012 (36 months), Luque-Fernandez 2013 (40 months), and Fatti 2012 (60 months); these results, however, could not be pooled as they had disparate time endpoints.

In terms of patient segmentation, such as demographics, setting (urban or rural), socioeconomic status and psychosocial indicators, these studies seldom demonstrated that one variable was significantly associated with outcome differences between control and intervention groups. None of the studies indicated that gender was significantly different between control and intervention groups. Luque-Fernandez was the only study which did not calculate the percentages of men and women separately in the control and intervention groups, but the overall percentage of women (70.6%) in the study was more than twice that of men (29.4%) [30]. Considering only the adult cohort studies and the control and intervention groups, the median age ranged from 31.7 years old in the intervention arm of CASA [26, 27] to 38 years old in the Tanzanian intervention arm of Mfinanga [31]. Among these adult cohort studies, the age differential between control and intervention arms were never stated to be significantly different. In the pediatric cohort study of Kheth’Impilo, there was no significant difference in median age between the control (6.2 years old) and the intervention (6.8 years old) groups [28]. In terms of the urban and rural setting of the studies, only CASA, Luque-Fernandez, Mfinanga, and Pearson were conducted in urban-only settings [24, 26, 27, 30, 31], while Franke was the only study conducted in a rural-only setting [25]. Studies which had both urban and rural sites were Braitstein and Kheth’Impilo [28, 29, 32]. In Braitstein, both control and intervention arms had a similar percentage of rural health centres (31% in intervention, and 30% in control) and the authors did not state that rural health centres significantly influenced the outcomes of interest [32]. In the adult cohort of Kheth’Impilo, rural facilities accounted for 5.1% of the intervention arm versus 6.5% of the control arm. Using survival analysis and the multiple imputation model for missing data, the adjusted hazard of mortality was significantly higher in rural areas than urban areas (aHR = 2.07, 95% CI 1.84 to 2.31), but the adjusted hazard of being LTFU was significantly lower in rural than urban areas (aHR = 0.62, 95% CI 0.53 to 0.71) [29]. For the pediatric cohort of Kheth’Impilo, there was actually a significant difference in rural facility percentage between intervention (2.8%) and control (10%) arms. Grimwood states that this significant difference however did not demonstrate a significant impact on mortality, and did not use rural facilities to analyse other outcomes of interest [28]. The intervention arm in Grimwood had significantly reduced mortality, but a non-significant decrease in LTFU (Figs 4 and 6). As for socioeconomic status, all included studies stated that their findings are relevant for resource-limited settings, although only CASA, Franke, and Pearson measured socioeconomic indicators [24–27]. In CASA, the intervention group actually had significantly higher unemployment, food scarcity, lack of basic services, and one-room households but with significantly lower substance abuse [26, 27]. Despite this, the intervention group still had increased retention, decrease mortality, and decreased LTFU or mortality at the end of two years, all of which were significant (Figs 3, 5 and 7). In Franke, the intervention arm showed worse access-to-care, literacy, depression, and mental and physical health indicators, but Franke adjusted for these indicators in the multivariate regression analysis [25]. The intervention arm in Franke only showed a significant improvement in LTFU or mortality (Fig 5). For Pearson, the authors state that there were no significant associations between adherence, and income, education, marital status, stigma, depression, or number of persons to whom patients disclosed. There were also no differences reported in stigma, self-efficacy, social support, or depression in the study [24]. In these studies, even if a patient segmentation variable was considered significantly different, none of the studies claimed that these variables were the main drivers of significant improvements in outcomes.

Finally, only three studies included cost data, Kheth’Impilo, Mfinanga, and Pearson [24, 28, 29, 31]. In Kheth’Impilo, where patient advocates made home visits weekly in the first month, and afterward between every one to three months, their reported average cost per patient per visit was USD 1.98, ranging from USD 1.88 to 3.43 [28, 29]. The Kheth’Impilo study was conducted from 2004 to 2010, and the report did not state which year was used as the reference, so it is difficult to adjust for inflation. For Mfinanga, which used 2012 USD prices, the lay-worker component of the one month, weekly home visits cost USD 14.74 in Tanzania and USD 13.03 in Zambia. These costs did not include the prices for the tuberculosis screening test (Gene Xpert) nor did they include the cryptococcal antigen test and the antifungal treatment. If these testing costs plus staff and clinic costs were all included, the full intervention cost per participant would be USD 67.26 in Tanzania and USD 54.19 in Zambia [31]. For Pearson, where patients came to the clinic for the DOT-plus intervention five days a week for six total weeks, the cost per participant for the entire six weeks was USD 33.00 [24]. That can then be divided by 30 total visits (during the six-week study period with five visits per week) for a price of USD 1.10 per patient visit. The study occurred from 2004 and 2006 but the article did not state which reference year was used for the price calculations. It seemed that of the studies that included costs, home visits could range from USD 1.98 [28, 29] to upwards of USD 14.74 [31] per patient, whereas each clinic visit cost USD 1.10 per patient [24] if inflation were not considered.

## Discussion

We identified two RCTs and five observational studies that evaluated supportive interventions to improve ART retention in LMIC; these studies evaluated four different types of interventions. Pearson (2007), one of two RCTs that we found, reported a significant increase in retention at 12 months for their six-week intervention of peer-delivered mDOT, which included psychosocial support, peer education on treatment and adherence, and strengthening the links between patients and clinic staff. Mfinanga (2015) also showed a significant improvement in retention at 12 months with their screening and treatment of cryptococcal infections and community-based adherence support intervention for patients with <200 CD4 cells/μL.

We also found that community-based interventions featuring a treatment or adherence supporter with home visits were effective in improving retention in care in resource-limited settings (Fatti 2012, Grimwood 2012, Mfinanga 2015, Muñoz 2010, Muñoz 2011, Pearson 2007). This rather modest benefit may have been due to high retention in care rates in the standard care arm (>73% in 6 of 7 trials) that results in lower power to detect differences. An adherence support group intervention also was associated with an 80% reduction in LTFU or death at 40 months (Luque-Fernandez 2013). Two interventions for patients at high risk of early mortality due to low CD4 counts at ART initiation were both effective in reducing LTFU and mortality [31, 32].

Six of the seven studies [24–29, 31] showed a significant improvement in retention, and all seven showed a significant decrease in the combined outcome of LTFU or mortality. Each study tested interventions with non-provider treatment or adherence supporters. Four studies included full or partial DOT or at least mentioned that the taking of medication was supervised [28, 29]. Studies featuring home visits embedded in community-based adherence/treatment supporter interventions showed significantly improved retention [25–29, 31].

Adherence clubs [30] and an extra nursing care intervention [32] also required patients to visit the clinic to receive the intervention. These interventions did not feature a component of home visits. Both Braitstein and Luque-Fernandez showed significantly decreased LTFU or mortality, despite Luque-Fernandez’s measuring the outcome at a time period more than three times longer (40 months) than that evaluated in Braitstein (318 days) [30, 32]. The fact that Luque-Fernandez only recruited participants who had been stable on ART for at least 18 months may have been a factor in its improved retention outcome over time [30]. The interventions evaluated in Braitstein and Luque-Fernandez continued to offer patients the features of the intervention for the duration of their studies [30, 32].

Taking small scale trials to scale nationally is fraught with difficulty, and understanding how to implement these interventions with fidelity at the national level is the next step in making use of these findings. Additionally, the costs of these interventions and the human resources required to implement them are mostly lacking in the studies we included in the review. Cost-effectiveness data will be needed to move the interventions to scale, particularly in an era of level resources.

As with any systematic review, our conclusions are limited only to the data we identified, which in turn is a function of the sensitivity of our search. To minimise this, we comprehensively searched five databases and hand searched for abstracts from the three main international HIV conferences and carefully reviewed the reference lists of all identified studies to assure completeness. We also used the GRADE system to judge the quality of the evidence for each intervention and outcome. The GRADE system has become the international standard for rating evidence for guidelines development [33], but there remain some issues with its use [34]. Due to the non-randomised designs of most of the included studies, inferring the causal relationship between the interventions and the outcomes may be hampered by confounding and the results may be more difficult to interpret compared to a systematic review involving only randomised trials. Thus, we separated Pearson from the pooled analysis of Franke and CASA, even though all studied the DOT-plus intervention measured at 12 months, to help reduce confounding. A final limitation is the geographical distribution of included studies, six of the seven included studies were conducted in Sub-Saharan Africa and one in Latin America, suggesting a need for interventions to be evaluated in other LMICs, particularly those where injection drug use may be the predominant form of transmission.

## Conclusion

While the quality of evidence ranged from very low to high, there seems to be consistent evidence that interventions focused on supportive interventions improve retention outcomes. In another systematic review, Nachega and colleagues have suggested that community-based interventions to improve adherence are successful because they help the patient build social networks, exercise more autonomy and reduce structural barriers, such as transport cost to the facility [35]. Based on the data in this review, multifaceted supportive interventions may be most appropriate for patients initiating ART because the patient advocate or treatment supporter actively visits the patient to engage them in care. For patients who are stable and on ART, adherence clubs may be a fitting approach to sustain retention in care while clinically high-risk patients may benefit from extra-nursing care. Moreover for future research, in order to more accurately see the effects of interventions to increase retention in care, it would be helpful for studies to track outcomes for years as the minority of included articles studied outcomes at three years or greater.

## Supporting information

S1 Table Core PubMed search strategy. Modified and adapted as needed for use in the other databases.
(PDF)

S1 File Risk of bias summary. (DOCX)

S2 File Brief summaries of the GRADE evidence quality. (DOCX)

S3 File Prisma checklist. (DOC)

## References

[1] United Nations Joint Programme on HIV/AIDS (UNAIDS). Fact Sheet—World AIDS Day 2017 Geneva, Switzerland: UNAIDS; 2017. Available from: http://www.unaids.org/en/resources/fact-sheet.

[2] Nakagawa F, May M, Phillips A. Life expectancy living with HIV: recent estimates and future implications. Current opinion in infectious diseases. 2013;26(1):17–25. Epub 2012/12/12. 10.1097/QCO.0b013e32835ba6b1 . 23221765 10.1097/QCO.0b013e32835ba6b1

[3] United Nations Joint Programme on HIV/AIDS (UNAIDS). Ending AIDS: Progress towards the 90-90-90 Targets. Geneva, Switzerland: UNAIDS, 2017.

[4] Phillips AN, Cambiano V, Nakagawa F, Revill P, Jordan MR, Hallett TB, et al Cost-effectiveness of public-health policy options in the presence of pretreatment NNRTI drug resistance in sub-Saharan Africa: a modelling study. The lancet HIV. 2017 Epub 2017/11/28. 10.1016/s2352-3018(17)30190-x . 29174084 10.1016/S2352-3018(17)30190-X PMC5843989

[5] Vandormael AM, Boulware DR, Tanser FC, Barnighausen TW, Stott KE, de Oliveira T. Brief Report: Virologic Monitoring Can Be a Cost-Effective Strategy to Diagnose Treatment Failure on First-Line ART. Journal of acquired immune deficiency syndromes (1999). 2016;71(4):462–6. Epub 2015/10/21. 10.1097/qai.0000000000000870 ; PubMed Central PMCID: PMCPMC4767659. 26484740 10.1097/QAI.0000000000000870 PMC4767659

[6] Anglemyer A, Rutherford GW, Horvath T, Baggaley RC, Egger M, Siegfried N. Antiretroviral therapy for prevention of HIV transmission in HIV-discordant couples. The Cochrane database of systematic reviews. 2013;4:CD009153 Epub 2013/05/02. 10.1002/14651858.CD009153.pub3 ; PubMed Central PMCID: PMCPMC4026368. 23633367 10.1002/14651858.CD009153.pub3 PMC4026368

[7] (WHO) WHO. Consolidated Guidelines on the Use of Antiretroviral Drugs for Treating and Preventing HIV Infection: What’s New. Policy Brief. Geneva, Switzerland: WHO, 2015.

[8] Rosen S, Fox MP, Gill CJ. Patient retention in antiretroviral therapy programs in sub-Saharan Africa: a systematic review. PLoS medicine. 2007;4(10):e298 Epub 2007/10/19. 10.1371/journal.pmed.0040298 ; PubMed Central PMCID: PMCPMC2020494. 17941716 10.1371/journal.pmed.0040298 PMC2020494

[9] Fox MP, Rosen S. Patient retention in antiretroviral therapy programs up to three years on treatment in sub-Saharan Africa, 2007–2009: systematic review. Tropical medicine & international health: TM & IH. 2010;15 Suppl 1:1–15. Epub 2010/07/14. 10.1111/j.1365-3156.2010.02508.x ; PubMed Central PMCID: PMCPMC2948795. 20586956 10.1111/j.1365-3156.2010.02508.x PMC2948795

[10] Fox MP, Rosen S. Systematic review of retention of pediatric patients on HIV treatment in low and middle-income countries 2008–2013. AIDS (London, England). 2015;29(4):493–502. Epub 2015/01/08. 10.1097/qad.0000000000000559 . 25565496 10.1097/QAD.0000000000000559

[11] Fox MP, Rosen S. Retention of adult patients on antiretroviral therapy in low- and middle-income countries: systematic review and meta-analysis 2008–2013. Journal of acquired immune deficiency syndromes (1999). 2015;69(1):98–108. Epub 2015/05/06. 10.1097/qai.0000000000000553 ; PubMed Central PMCID: PMCPMC4422218. 25942461 10.1097/QAI.0000000000000553 PMC4422218

[12] Mitiku I, Arefayne M, Mesfin Y, Gizaw M. Factors associated with loss to follow-up among women in Option B+ PMTCT programme in northeast Ethiopia: a retrospective cohort study. Journal of the International AIDS Society. 2016;19(1):20662 Epub 2016/03/24. 10.7448/IAS.19.1.20662 ; PubMed Central PMCID: PMCPMC4803835. 27005750 10.7448/IAS.19.1.20662 PMC4803835

[13] Tenthani L, Haas AD, Tweya H, Jahn A, van Oosterhout JJ, Chimbwandira F, et al Retention in care under universal antiretroviral therapy for HIV-infected pregnant and breastfeeding women ('Option B+') in Malawi. AIDS (London, England). 2014;28(4):589–98. Epub 2014/01/29. 10.1097/qad.0000000000000143 ; PubMed Central PMCID: PMCPMC4009400. 24468999 10.1097/QAD.0000000000000143 PMC4009400

[14] Haas AD, Tenthani L, Msukwa MT, Tal K, Jahn A, Gadabu OJ, et al Retention in care during the first 3 years of antiretroviral therapy for women in Malawi's option B+ programme: an observational cohort study. The lancet HIV. 2016;3(4):e175–82. Epub 2016/04/03. 10.1016/S2352-3018(16)00008-4 . 27036993 10.1016/S2352-3018(16)00008-4 PMC4904064

[15] World Bank. Country and Lending Groups: World Bank; 2016 [12 May 2014]. Available from: http://data.worldbank.org/about/country-and-lending-groups-Lower_middle_income.

[16] Kredo T, Ford N, Adeniyi FB, Garner P. Decentralising HIV treatment in lower- and middle-income countries. The Cochrane database of systematic reviews. 2013;6:CD009987 Epub 2013/06/29. 10.1002/14651858.CD009987.pub2 . 23807693 10.1002/14651858.CD009987.pub2 PMC10009870

[17] Kredo T, Adeniyi FB, Bateganya M, Pienaar ED. Task shifting from doctors to non-doctors for initiation and maintenance of antiretroviral therapy. The Cochrane database of systematic reviews. 2014;7:CD007331 Epub 2014/07/02. 10.1002/14651858.CD007331.pub3 . 24980859 10.1002/14651858.CD007331.pub3 PMC11214583

[18] Mbeye NM, Adetokunboh O, Negussie E, Kredo T, Wiysonge CS. Shifting tasks from pharmacy to non-pharmacy personnel for providing antiretroviral therapy to people living with HIV: a systematic review and meta-analysis. BMJ open. 2017;7(8):e015072 Epub 2017/08/31. 10.1136/bmjopen-2016-015072 ; PubMed Central PMCID: PMCPMC5724105. 28851770 10.1136/bmjopen-2016-015072 PMC5724105

[19] Higgins JP, Green S. Cochrane handbook for systematic reviews of interventions. Chicester, UK: Wiley Online Library; 2008.

[20] Denison JA, O'Reilly KR, Schmid GP, Kennedy CE, Sweat MD. HIV voluntary counseling and testing and behavioral risk reduction in developing countries: a meta-analysis, 1990–2005. AIDS and behavior. 2008;12(3):363–73. Epub 2007/12/28. 10.1007/s10461-007-9349-x . 18161018 10.1007/s10461-007-9349-x

[21] Kennedy C, O'Reilly K, Medley A, Sweat M. The impact of HIV treatment on risk behaviour in developing countries: a systematic review. AIDS care. 2007;19(6):707–20. Epub 2007/06/19. 10.1080/09540120701203261 . 17573590 10.1080/09540120701203261

[22] Kennedy CE, Spaulding AB, Brickley DB, Almers L, Mirjahangir J, Packel L, et al Linking sexual and reproductive health and HIV interventions: a systematic review. Journal of the International AIDS Society. 2010;13:26 Epub 2010/07/21. 10.1186/1758-2652-13-26 ; PubMed Central PMCID: PMCPMC2918569. 20642843 10.1186/1758-2652-13-26 PMC2918569

[23] Guyatt G, Oxman AD, Akl EA, Kunz R, Vist G, Brozek J, et al GRADE guidelines: 1. Introduction-GRADE evidence profiles and summary of findings tables. Journal of clinical epidemiology. 2011;64(4):383–94. Epub 2011/01/05. 10.1016/j.jclinepi.2010.04.026 . 21195583 10.1016/j.jclinepi.2010.04.026

[24] Pearson CR, Micek MA, Simoni JM, Hoff PD, Matediana E, Martin DP, et al Randomized control trial of peer-delivered, modified directly observed therapy for HAART in Mozambique. Journal of acquired immune deficiency syndromes (1999). 2007;46(2):238–44. Epub 2007/08/19. 10.1097/QAI.0b013e318153f7ba ; PubMed Central PMCID: PMCPMC4044044. 17693890 10.1097/QAI.0b013e318153f7ba PMC4044044

[25] Franke MF, Kaigamba F, Socci AR, Hakizamungu M, Patel A, Bagiruwigize E, et al Improved retention associated with community-based accompaniment for antiretroviral therapy delivery in rural Rwanda. Clinical infectious diseases: an official publication of the Infectious Diseases Society of America. 2013;56(9):1319–26. Epub 2012/12/20. 10.1093/cid/cis1193 . 23249611 10.1093/cid/cis1193

[26] Munoz M, Finnegan K, Zeladita J, Caldas A, Sanchez E, Callacna M, et al Community-based DOT-HAART accompaniment in an urban resource-poor setting. AIDS and behavior. 2010;14(3):721–30. Epub 2009/04/17. 10.1007/s10461-009-9559-5 . 19370409 10.1007/s10461-009-9559-5 PMC8327366

[27] Munoz M, Bayona J, Sanchez E, Arevalo J, Sebastian JL, Arteaga F, et al Matching social support to individual needs: a community-based intervention to improve HIV treatment adherence in a resource-poor setting. AIDS and behavior. 2011;15(7):1454–64. Epub 2010/04/13. 10.1007/s10461-010-9697-9 . 20383572 10.1007/s10461-010-9697-9 PMC6942493

[28] Grimwood A, Fatti G, Mothibi E, Malahlela M, Shea J, Eley B. Community adherence support improves programme retention in children on antiretroviral treatment: a multicentre cohort study in South Africa. Journal of the International AIDS Society. 2012;15(2):17381 Epub 2012/06/21. 10.7448/IAS.15.2.17381 ; PubMed Central PMCID: PMCPMC3499784. 22713255 10.7448/IAS.15.2.17381 PMC3499784

[29] Fatti G, Meintjes G, Shea J, Eley B, Grimwood A. Improved survival and antiretroviral treatment outcomes in adults receiving community-based adherence support: 5-year results from a multicentre cohort study in South Africa. Journal of acquired immune deficiency syndromes (1999). 2012;61(4):e50–8. Epub 2012/07/31. 10.1097/QAI.0b013e31826a6aee . 22842842 10.1097/QAI.0b013e31826a6aee

[30] Luque-Fernandez MA, Van Cutsem G, Goemaere E, Hilderbrand K, Schomaker M, Mantangana N, et al Effectiveness of patient adherence groups as a model of care for stable patients on antiretroviral therapy in Khayelitsha, Cape Town, South Africa. PloS one. 2013;8(2):e56088 Epub 2013/02/19. 10.1371/journal.pone.0056088 ; PubMed Central PMCID: PMCPMC3571960. 23418518 10.1371/journal.pone.0056088 PMC3571960

[31] Mfinanga S, Chanda D, Kivuyo SL, Guinness L, Bottomley C, Simms V, et al Cryptococcal meningitis screening and community-based early adherence support in people with advanced HIV infection starting antiretroviral therapy in Tanzania and Zambia: an open-label, randomised controlled trial. Lancet (London, England). 2015;385(9983):2173–82. Epub 2015/03/15. 10.1016/s0140-6736(15)60164-7 . 25765698 10.1016/S0140-6736(15)60164-7

[32] Braitstein P, Siika A, Hogan J, Kosgei R, Sang E, Sidle J, et al A clinician-nurse model to reduce early mortality and increase clinic retention among high-risk HIV-infected patients initiating combination antiretroviral treatment. Journal of the International AIDS Society. 2012;15(1):7 Epub 2012/02/22. 10.1186/1758-2652-15-7 ; PubMed Central PMCID: PMCPMC3297518. 22340703 10.1186/1758-2652-15-7 PMC3297518

[33] World Health Organization (WHO). WHO Handbook for Guideline Development. Geneva, Switzerland: WHO, 2014.

[34] Sinclair D, Isba R, Kredo T, Zani B, Smith H, Garner P. World Health Organization guideline development: an evaluation. PloS one. 2013;8(5):e63715 Epub 2013/06/07. 10.1371/journal.pone.0063715 ; PubMed Central PMCID: PMCPMC3669321. 23741299 10.1371/journal.pone.0063715 PMC3669321

[35] Nachega JB, Adetokunboh O, Uthman OA, Knowlton AW, Altice FL, Schechter M, et al Community-Based Interventions to Improve and Sustain Antiretroviral Therapy Adherence, Retention in HIV Care and Clinical Outcomes in Low- and Middle-Income Countries for Achieving the UNAIDS 90-90-90 Targets. Current HIV/AIDS reports. 2016 Epub 2016/08/01. 10.1007/s11904-016-0325-9 . 27475643 10.1007/s11904-016-0325-9 PMC5357578

