# Supplementary material for: Supportive interventions to improve retention on ART in people with HIV in low- and middle-income countries: A systematic review
Source: PLoS One. 2018 Dec 14;13(12):e0208814. doi: 10.1371/journal.pone.0208814 (PMC6294385; doi:10.1371/journal.pone.0208814)
Supplement: S1 Table — Modified and adapted as needed for use in the other databases. (PDF) [file pone.0208814.s001.pdf]

S1 Table: Core PubMed search strategy. Modified and adapted as needed for use in the other databases.

Search	PubMed Query
#7	**#6 AND date range 2015-04-27 to 2017-04-04**
#6	**#1 AND #2 AND #3 AND #4 AND #5**
#5	Search Afghanistan[tiab] OR Albania[tiab] OR Algeria[tiab] OR American Samoa[tiab] OR Angola[tiab] OR Antigua and Barbuda[tiab] OR Argentina[tiab] OR Armenia[tiab] OR Azerbaijan[tiab] OR Bangladesh[tiab] OR Belarus[tiab] OR Belize[tiab] OR Benin[tiab] OR Bhutan[tiab] OR Bolivia[tiab] OR Bosnia and Herzegovina[tiab] OR Botswana[tiab] OR Brazil[tiab] OR Bulgaria[tiab] OR Burkina Faso[tiab] OR Burundi[tiab] OR Cambodia[tiab] OR Cameroon[tiab] OR Cabo Verde[tiab] OR Central African Republic[tiab] OR Chad[tiab] OR Chile[tiab] OR China[tiab] OR Colombia[tiab] OR Comoros[tiab] OR OR Congo[tiab] OR Costa Rica[tiab] OR Côte d'Ivoire[tiab] OR Cuba[tiab] OR Djibouti[tiab] OR Dominica[tiab] OR Dominican Republic[tiab] OR Ecuador[tiab] OR Egypt[tiab] OR El Salvador[tiab] OR Eritrea[tiab] OR Ethiopia[tiab] OR Fiji[tiab] OR Gabon[tiab] OR Gambia[tiab] OR Georgia[tiab] OR Ghana[tiab] OR Grenada[tiab] OR Guatemala[tiab] OR Guinea[tiab] OR Guinea-Bissau[tiab] OR Guyana[tiab] OR Haiti[tiab] OR Honduras[tiab] OR India[tiab] OR Indonesia[tiab] OR Iran[tiab] OR Iraq[tiab] OR Jamaica[tiab] OR Jordan[tiab] OR Kazakhstan[tiab] OR Kenya[tiab] OR Kiribati[tiab] OR North Korea [tiab] OR Kosovo[tiab] OR Kyrgyz [tiab] OR Lao[tiab] OR Latvia[tiab] OR Lebanon[tiab] OR Lesotho[tiab] OR Liberia[tiab] OR Libya[tiab] OR Lithuania[tiab] OR Macedonia[tiab] OR Madagascar[tiab] OR Malawi[tiab] OR Malaysia[tiab] OR Maldives[tiab] OR Mali[tiab] OR Marshall Islands[tiab] OR Mauritania[tiab] OR Mauritius[tiab] OR Mexico[tiab] OR Micronesia[tiab] OR Moldova[tiab] OR Mongolia[tiab] OR Montenegro[tiab] OR Morocco[tiab] OR Mozambique[tiab] OR Myanmar[tiab] OR Namibia[tiab] OR Nepal[tiab] OR Nicaragua[tiab] OR Niger[tiab] OR Nigeria[tiab] OR Pakistan[tiab] OR Palau[tiab] OR Panama[tiab] OR Papua New Guinea[tiab] OR Paraguay[tiab] OR Peru[tiab] OR Philippines[tiab] OR Romania[tiab] OR Russia[tiab] OR Russian Federation[tiab] OR Rwanda[tiab] OR Samoa[tiab] OR Sao Tome[tiab] OR Senegal[tiab] OR Serbia[tiab] OR Seychelles[tiab] OR Sierra Leone[tiab] OR Solomon Islands[tiab] OR Somalia[tiab] OR South Africa[tiab] OR South Sudan[tiab] OR Sri Lanka[tiab] OR St. Lucia[tiab] OR St. Vincent[tiab] OR Grenadines[tiab] OR Sudan[tiab] OR Suriname[tiab] OR Swaziland[tiab] OR Syrian Arab Republic[tiab] OR Tajikistan[tiab] OR Tanzania[tiab] OR Thailand[tiab] OR Timor-Leste[tiab] OR Togo[tiab] OR Tonga[tiab] OR Tunisia[tiab] OR Turkey[tiab] OR Turkmenistan[tiab] OR Tuvalu[tiab] OR Uganda[tiab] OR Ukraine[tiab] OR Uruguay[tiab] OR Uzbekistan[tiab] OR Vanuatu[tiab] OR Venezuela[tiab] OR Vietnam[tiab] OR West Bank[tiab] OR Gaza[tiab] OR Yemen[tiab] OR Zambia[tiab] OR Zimbabwe[tiab] OR Afghanistan[mh] OR Albania[mh] OR Algeria[mh] OR American Samoa[mh] OR Angola[mh] OR Antigua and Barbuda[mh] OR Argentina[mh] OR Armenia[mh] OR Azerbaijan[mh] OR Bangladesh[mh] OR Belarus[mh] OR Belize[mh] OR Benin[mh] OR Bhutan[mh] OR Bolivia[mh] OR Bosnia and Herzegovina[mh] OR Botswana[mh] OR Brazil[mh] OR Bulgaria[mh] OR Burkina Faso[mh] OR Burundi[mh] OR Cambodia[mh] OR Cameroon[mh] OR Cabo Verde[mh] OR Central African Republic[mh] OR Chad[mh] OR Chile[mh] OR China[mh] OR Colombia[mh] OR Comoros[mh] OR OR Congo[mh] OR Costa Rica[mh] OR Côte d'Ivoire[mh] OR Cuba[mh] OR Djibouti[mh] OR Dominica[mh] OR Dominican Republic[mh] OR Ecuador[mh] OR Egypt[mh] OR El Salvador[mh] OR Eritrea[mh] OR Ethiopia[mh] OR Fiji[mh] OR Gabon[mh] OR Gambia[mh] OR Georgia[mh]

	OR Ghana[mh] OR Grenada[mh] OR Guatemala[mh] OR Guinea[mh] OR Guinea-Bissau[mh] OR Guyana[mh] OR Haiti[mh] OR Honduras[mh] OR India[mh] OR Indonesia[mh] OR Iran[mh] OR Iraq[mh] OR Jamaica[mh] OR Jordan[mh] OR Kazakhstan[mh] OR Kenya[mh] OR Kiribati[mh] OR North Korea [mh] OR Kosovo[mh] OR Kyrgyz [mh] OR Lao[mh] OR Latvia[mh] OR Lebanon[mh] OR Lesotho[mh] OR Liberia[mh] OR Libya[mh] OR Lithuania[mh] OR Macedonia[mh] OR Madagascar[mh] OR Malawi[mh] OR Malaysia[mh] OR Maldives[mh] OR Mali[mh] OR Marshall Islands[mh] OR Mauritania[mh] OR Mauritius[mh] OR Mexico[mh] OR Micronesia[mh] OR Moldova[mh] OR Mongolia[mh] OR Montenegro[mh] OR Morocco[mh] OR Mozambique[mh] OR Myanmar[mh] OR Namibia[mh] OR Nepal[mh] OR Nicaragua[mh] OR Niger[mh] OR Nigeria[mh] OR Pakistan[mh] OR Palau[mh] OR Panama[mh] OR Papua New Guinea[mh] OR Paraguay[mh] OR Peru[mh] OR Philippines[mh] OR Romania[mh] OR Russia[mh] OR Russian Federation[mh] OR Rwanda[mh] OR Samoa[mh] OR Sao Tome[mh] OR Senegal[mh] OR Serbia[mh] OR Seychelles[mh] OR Sierra Leone[mh] OR Solomon Islands[mh] OR Somalia[mh] OR South Africa[mh] OR South Sudan[mh] OR Sri Lanka[mh] OR St. Lucia[mh] OR St. Vincent[mh] OR Grenadines[mh] OR Sudan[mh] OR Suriname[mh] OR Swaziland[mh] OR Syrian Arab Republic[mh] OR Tajikistan[mh] OR Tanzania[mh] OR Thailand[mh] OR Timor-Leste[mh] OR Togo[mh] OR Tonga[mh] OR Tunisia[mh] OR Turkey[mh] OR Turkmenistan[mh] OR Tuvalu[mh] OR Uganda[mh] OR Ukraine[mh] OR Uruguay[mh] OR Uzbekistan[mh] OR Vanuatu[mh] OR Venezuela[mh] OR Vietnam[mh] OR West Bank[mh] OR Gaza[mh] OR Yemen[mh] OR Zambia[mh] OR Zimbabwe[mh]
#4	Search (randomized controlled trial[pt] OR controlled clinical trial[pt] OR randomized controlled trials[MeSH] OR random allocation[MeSH] OR random*[tiab] OR cohort*[tiab] OR Cohort studies[MeSH] OR observational[tiab]) NOT (animals[MeSH] NOT human[MeSH])
#3	Search (retention[tiab] OR retain*[tiab] OR "lost to follow-up"[tiab] OR "loss to follow-up"[tiab] OR ("loss*"[tiab] AND "follow up"[tiab]) OR LTFU[tiab] OR attrition[tiab] OR "loss to care"[tiab] OR "lost to care"[tiab] OR "loss to program*"[tiab] OR "lost to program*"[tiab] OR default*[tiab] OR engage*[tiab] OR disengage*[tiab])
#2	Search (HAART[tiab] OR ART[tiab] OR cART[tiab] OR antiretroviral[tiab] OR anti-retroviral[tiab] OR anti-viral[tiab] OR antiviral[tiab] OR "Antiretroviral Therapy, Highly Active"[Mesh])
#1	Search (HIV Infections[MeSH] OR HIV[MeSH] OR HIV*[tiab] OR hiv-1[tiab] OR hiv-2*[tiab] OR hiv1[tiab] OR hiv2[tiab] OR hiv infect*[tiab] OR human immunodeficiency virus[tiab]OR human immune deficiency virus[tiab] OR human immuno-deficiency virus[tiab] OR human immune-deficiency virus[tiab] OR ((human immun*) AND(deficiency virus[tiab])) OR acquired immunodeficiency syndrome*[tiab] OR acquired immune deficiency syndrome*[tiab] OR acquired immuno-deficiency syndrome*[tiab] OR acquired immune-deficiency syndrome*[tiab] OR ((acquired immun*) AND (deficiency syndrome[tiab])) or “sexually transmitted diseases, viral”[mh])
