# Supplementary material for: Supportive interventions to improve retention on ART in people with HIV in low- and middle-income countries: A systematic review
Source: PLoS One. 2018 Dec 14;13(12):e0208814. doi: 10.1371/journal.pone.0208814 (PMC6294385; doi:10.1371/journal.pone.0208814)
Supplement: S1 File — (DOCX) [file pone.0208814.s002.docx]

**S1 File.** **Risk of bias summary.**

**Review authors' judgments about each risk of bias item for each included study**


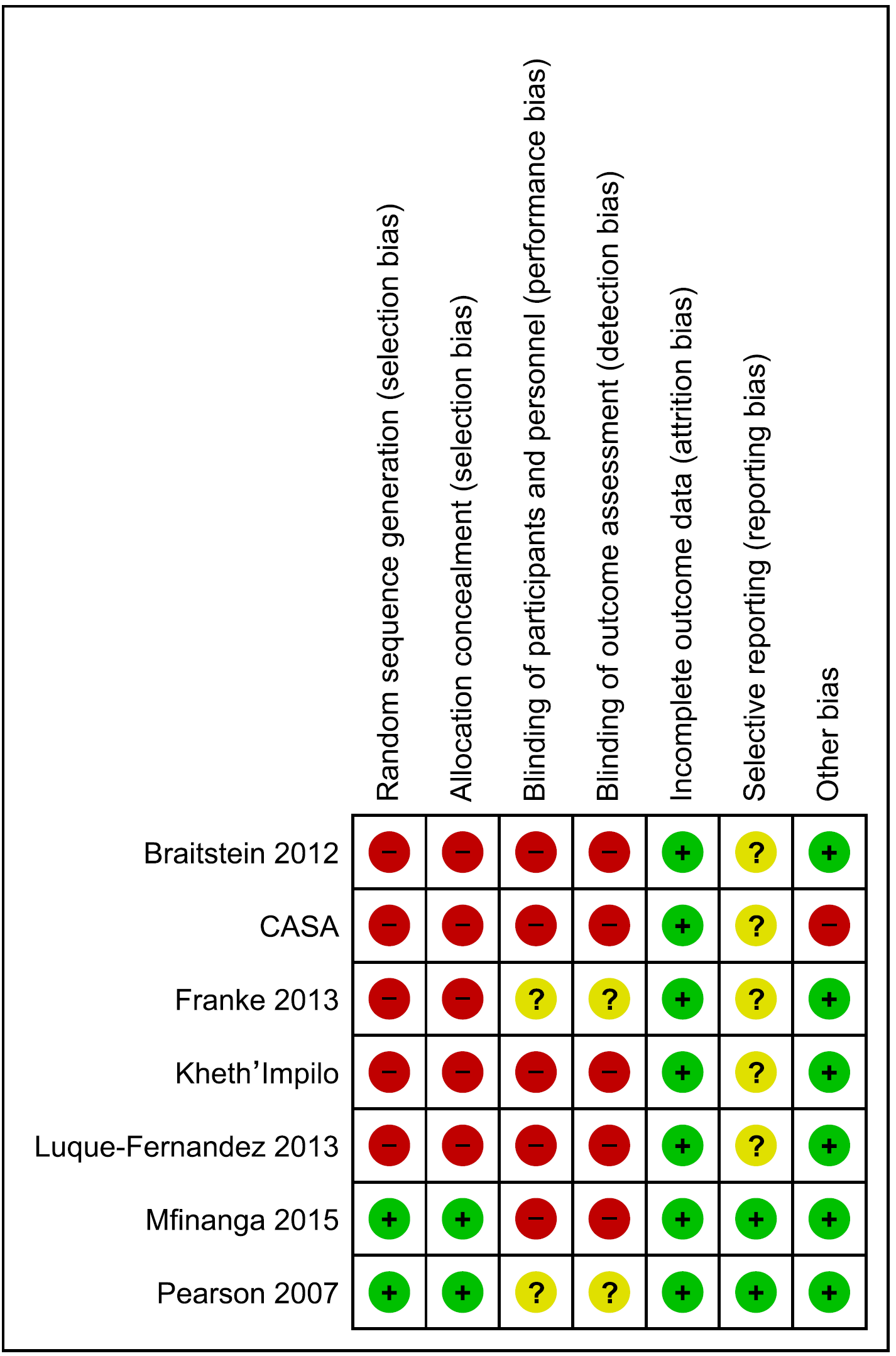


**Summary of bias judgment**

**
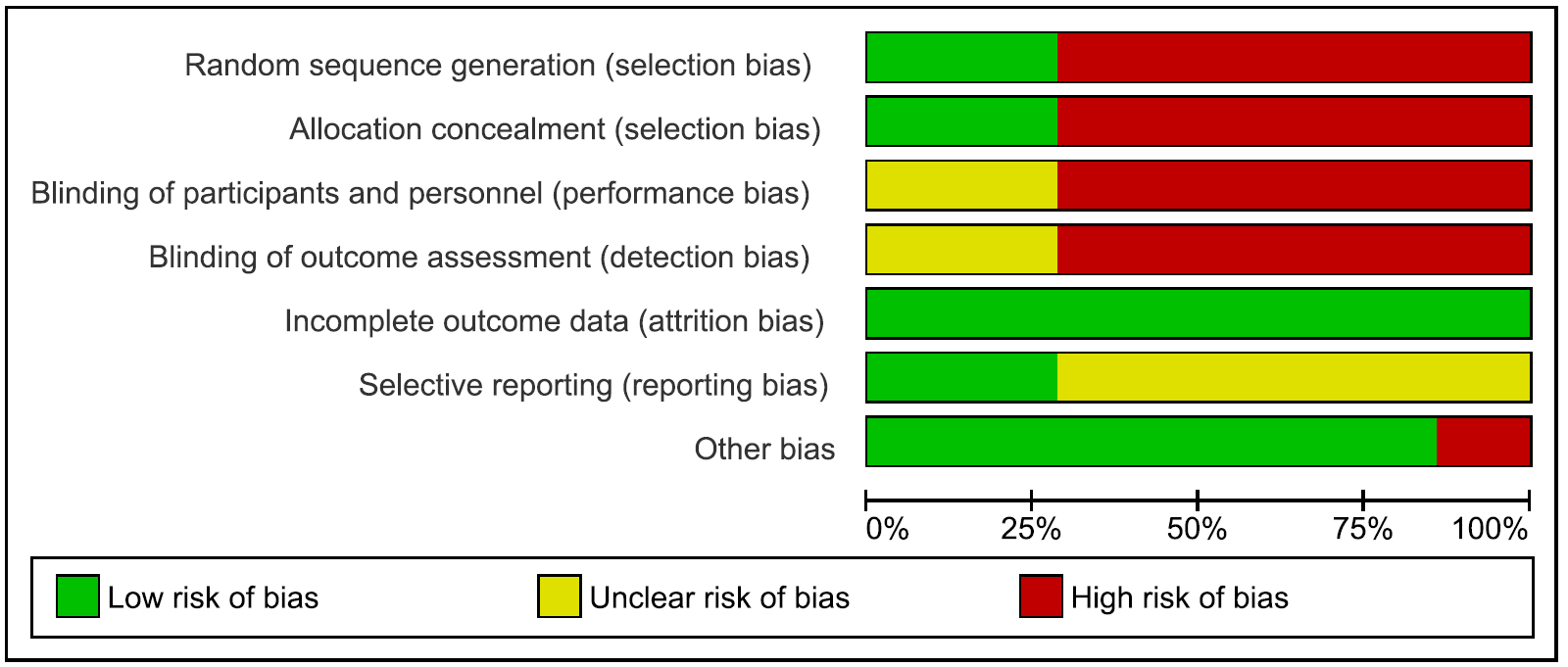
**

**Rigor of included observational studies**


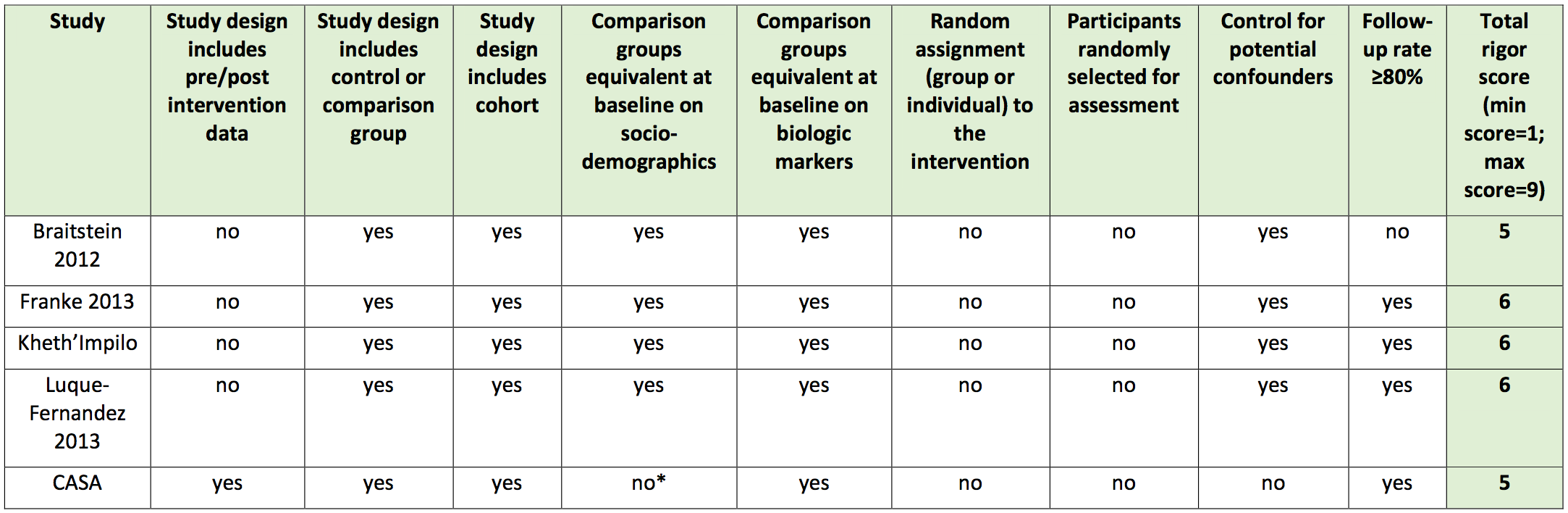


*Worse in intervention group
