# Supplementary material for: Supportive interventions to improve retention on ART in people with HIV in low- and middle-income countries: A systematic review
Source: PLoS One. 2018 Dec 14;13(12):e0208814. doi: 10.1371/journal.pone.0208814 (PMC6294385; doi:10.1371/journal.pone.0208814)
Supplement: S2 File — (DOCX) [file pone.0208814.s003.docx]

**S2 Table. Brief summaries of the GRADE evidence quality.**

**DOT-plus vs. usual care**

**Randomised controlled trial (adult population):**

- There is moderate quality evidence from one trial (Pearson 2007) that the use of a DOT-plus intervention improved retention in adults on ART at 12 months (RR 1.14, 95% CI 1.02 to 1.27). Evidence quality was graded down one level for serious risk of bias (lack of blinding).

**Observational cohort studies (adult population):**

- There is very low quality evidence from one study (CASA) that the use of a DOT-plus intervention reduced mortality in adults on ART at 24 months (RR 0.35, 95% CI 0.15 to 0.83). Evidence quality was graded down two levels for very serious imprecision (very few events). Due to this imprecision, it was not appropriate to grade up for large effect.
- There is very low quality evidence in pooled data from two studies (CASA and Franke 2013) that the use of a DOT-plus intervention reduced mortality in adults on ART at 12 months (RR 0.52, 95% CI 0.30 to 0.87). Evidence quality was graded down one level for serious imprecision (few events) and one level for serious inconsistency (marked difference in effect size).
- There is low quality evidence in pooled data from two studies (CASA and Franke 2013) that the use of a DOT-plus intervention improved program retention in adults on ART at 12 months (RR 1.10, 95% CI 1.04 to 1.16). It was not necessary to adjust evidence quality.
- There is very low quality evidence from one study (Franke 2013) that the use of a DOT-plus intervention did not significantly reduce losses to follow-up in adults on ART at 12 months (RR 0.30, 95% CI 0.08 to 1.09). Evidence quality was graded down two levels for very serious imprecision (very few events).

**Community-based adherence support vs. usual care**

**Observational cohort studies (adult population):**

- There is low quality evidence from one study (Kheth’Impilo) that the use of a multi-faceted community-based supportive intervention reduced mortality in adults on ART at 60 months (RR 0.85, 95% CI 0.81 to 0.89). It was not necessary to adjust evidence quality.
- There is low quality evidence from one study (Kheth’Impilo) that the use of a multi-faceted community-based supportive intervention improved program retention in adults on ART at 60 months (RR 1.07, 95% CI 1.07 to 1.08). It was not necessary to adjust evidence quality.
- There is very low quality evidence from one study (CASA) that the use of a multi-faceted community-based supportive intervention improved program retention in adults on ART at 24 months (RR 1.68, 95% CI 1.29 to 2.18). Evidence quality was graded down one level for serious imprecision (few events).
- There is low quality evidence from one study (Kheth’Impilo) that the use of a multi-faceted community-based supportive intervention reduced loss to follow-up in adults on ART at 60 months (RR 0.75, 95% CI 0.72 to 0.78). It was not necessary to adjust evidence quality.

**Observational study (paediatric population):**

- There is moderate quality evidence from one study (Kheth’Impilo) that the use of a multi-faceted community-based supportive intervention reduced mortality in children on ART at 36 months (RR 0.46, 95% CI 0.26 to 0.82). Evidence quality was graded up one level due to the large effect, given the absence of other downgrading.
- There is low quality evidence from one study (Kheth’Impilo) that the use of a multi-faceted community-based supportive intervention improved program retention in children on ART at 36 months (RR 1.07, 95% CI 1.03 to 1.11). It was not necessary to adjust evidence quality.
- There is low quality evidence from one study (Kheth’Impilo) that the use of a multi-faceted community-based supportive intervention did not significantly reduce losses to follow-up in children on ART at 36 months (RR 0.82, 95% CI 0.50 to 1.38). It was not necessary to adjust evidence quality.

**Adherence club intervention vs. usual care**

**Observational cohort study (adult population)**

- There is very low quality evidence from one study (Luque-Fernandez 2013) that the use of “adherence clubs” improved program retention in adults on ART at 40 months (RR 1.14, 95% CI 1.11 to 1.17). Evidence quality was graded down one level for indirectness (retrospective analysis).

**Extra care for high-risk patients vs. usual care**

**Randomised controlled trial (adult population):**

- There is high quality evidence from one trial (Mfinanga 2015) that providing extra care to high-risk patients reduced mortality in adults on ART at 12 months (RR 0.74, 95% CI 0.60 to 0.91). It was not necessary to adjust evidence quality.
- There is moderate quality evidence from one trial (Mfinanga 2015) that providing extra care to high-risk patients improved program retention in adults on ART at 12 months (RR 1.06, 95% CI 1.01 to 1.10). Evidence quality was graded down one level for serious risk of bias (lack of blinding).
- There is low quality evidence from one trial (Mfinanga 2015) that providing extra care to high-risk patients did not significantly reduce losses to follow-up in adults on ART at 12 months (RR 1.04, 95% CI 0.60 to 1.81). Evidence quality was graded down one level for serious risk of bias (lack of blinding) and one level for serious imprecision (few events).

**Observational cohort study (adult population):**

- There is very low quality evidence from one study (Braitstein 2012) that providing extra care to high-risk patients reduced mortality in adults on ART at 10 months (RR 0.69, 95% CI 0.50 to 0.94). Evidence quality was graded down one level for indirectness (retrospective analysis).
- There is very low quality evidence from one study (Braitstein 2012) that providing extra care to high-risk patients improved program retention in adults on ART at 10 months (RR 1.14, 95% CI 1.08 to 1.20). Evidence quality was graded down one level for indirectness (retrospective analysis).
- There is very low quality evidence from one study (Braitstein 2012) that providing extra care to high-risk patients reduced loss to follow-up in adults on ART at 10 months (RR 0.78, 95% CI 0.67 to 0.92). Evidence quality was graded down one level for indirectness (retrospective analysis).
- There is very low quality evidence from one study (Braitstein 2012) that providing extra care to high-risk patients reduced loss to follow-up or death in adults on ART at 10 months (RR 0.76, 95% CI 0.66 to 0.87). Evidence quality was graded down one level for indirectness (retrospective analysis).
